# The automatic recognition and counting of cough

**DOI:** 10.1186/1745-9974-2-8

**Published:** 2006-09-28

**Authors:** Samantha J Barry, Adrie D Dane, Alyn H Morice, Anthony D Walmsley

**Affiliations:** 1 Department of Chemistry, Faculty of Science and the Environment, University of Hull, Cottingham Road, Hull, HU6 7RX, UK; 2 Department of Academic Medicine, University of Hull, Cottingham Road, Hull, HU6 7RX, UK

## Abstract

**Background:**

Cough recordings have been undertaken for many years but the analysis of cough frequency and the temporal relation to trigger factors have proven problematic. Because cough is episodic, data collection over many hours is required, along with real-time aural analysis which is equally time-consuming.

A method has been developed for the automatic recognition and counting of coughs in sound recordings.

**Methods:**

The Hull Automatic Cough Counter (HACC) is a program developed for the analysis of digital audio recordings. HACC uses digital signal processing (DSP) to calculate characteristic spectral coefficients of sound events, which are then classified into cough and non-cough events by the use of a probabilistic neural network (PNN). Parameters such as the total number of coughs and cough frequency as a function of time can be calculated from the results of the audio processing.

Thirty three smoking subjects, 20 male and 13 female aged between 20 and 54 with a chronic troublesome cough were studied in the hour after rising using audio recordings.

**Results:**

Using the graphical user interface (GUI), counting the number of coughs identified by HACC in an hour long recording, took an average of 1 minute 35 seconds, a 97.5% reduction in counting time. HACC achieved a sensitivity of 80% and a specificity of 96%. Reproducibility of repeated HACC analysis is 100%.

**Conclusion:**

An automated system for the analysis of sound files containing coughs and other non-cough events has been developed, with a high robustness and good degree of accuracy towards the number of actual coughs in the audio recording.

## Background

Cough is the commonest symptom for which patients seek medical advice [1]. Population studies reported prevalence of cough to vary between 3% and 40% [2-4]. As cough affects us all, its management has massive health economic consequences with the use of over-the-counter cough remedies in the UK being estimated at 75 million sales per annum [5]. Cough is conventionally considered to consist of an initial deep inspiration followed by expiration against a closed glottis that then opens [6-8]. As a result a characteristic phonation is formed, which is composed of two distinct components termed first and second cough sounds [6,7].

Whilst the recognition of a single cough event is relatively easy, the assessment of cough frequency over a long period of time remains difficult both for clinical and research purposes. Part of the problem is the paroxysmal nature of cough necessitating recording over a prolonged time period in order to generate an accurate estimate of cough frequency. Subjective recording or scoring of cough is unreliable as individual perception of cough differs from mild irritation to marked impairment of quality of life [9,10]. In addition, subjective assessment of cough frequency during the night-time has been shown to be unreliable [11,12]. The simple recording of cough sound using a microphone and cassette recorder allows for counting of the cough events, however, analysis is very time consuming even with the application of sound activated recording or methods for removing silence [7,8,13,14]. Similarly, the use of cough recorders that incorporate electromyogram (EMG) [15,16] or modified Holter monitor [17,18] require manual reading of the recorded tapes by a trained investigator. Automatic cough recognition from ambulatory multi-channel physiological recordings have been reported [19]. Here we describe a method for automatic recognition and counting of coughs solely from sound recordings which reduces the processing time and removes the need for trained listeners.

## Materials and methods

The method, Hull Automatic Cough Counter (HACC) operates in three steps.

Firstly, the signal is analysed to identify periods of sound within the recordings; these sound events are then extracted and any periods of silence are omitted from further analysis. Secondly, digital signal processing (DSP) is applied to calculate the characteristic feature vectors which represent each sound event. The techniques used are linear predictive coding (LPC) and a bank-of-filters front-end processor. The resultant coefficients are reduced by principal component analysis (PCA); this step highlights the components of the data that contain the most variance, such that only these components are used for further analysis. Thirdly, the sound events are then classified into cough and non-cough events by use of a probabilistic neural network (PNN) [20]. The PNN is trained to recognise the feature vectors of reference coughs and non-coughs and classify future sound events appropriately.

Parameters such as the total number of coughs and cough frequency as a function of time can be calculated from the results of the audio processing. Currently, the determination of the number of coughs inside each cough event is carried out by a human listener.

### Subjects and sound recording

Thirty three smoking subjects, 20 male and 13 female aged between 20 and 54 with a chronic troublesome cough were studied in the hour after rising. The smoking histories of the subjects ranged between 5 and 100 pack years with a mean of 21.4. As part of a previously published controlled trial [21] a cigarette was administered 20 minutes after the start of recording. All the subjects were studied in the outpatients clinic with the subjects ambulatory and television and conversation freely permitted.

Sound was recorded at a sampling frequency of 48 kHz using a Sony ECM-TIS Lapel microphone connected to a Sony TCD-D8 Walkman DAT-recorder. For each of the subjects, this recording was converted into 44.1 kHz 16 bit mono Microsoft wave format. To minimise data storage the sound recordings are initially analysed at a sampling frequency *f*_s _of 11.025 kHz by using only every fourth point.

### Software and hardware

All software was developed under Matlab^® ^version 6.1 [22]. The following Matlab toolboxes were used: PLS_Toolbox version 2.1.1 [23], Signal processing toolbox version 5.1 [24], Neural network toolbox version 4.0.1 [25] and Voicebox (a free toolbox for speech recognition) [26]. The programs were executed under Windows 2000 on a 1.4 GHz Pentium 4 PC with 256 megabytes of RAM.

### Determination and classification of sound events

**Figure 1:**
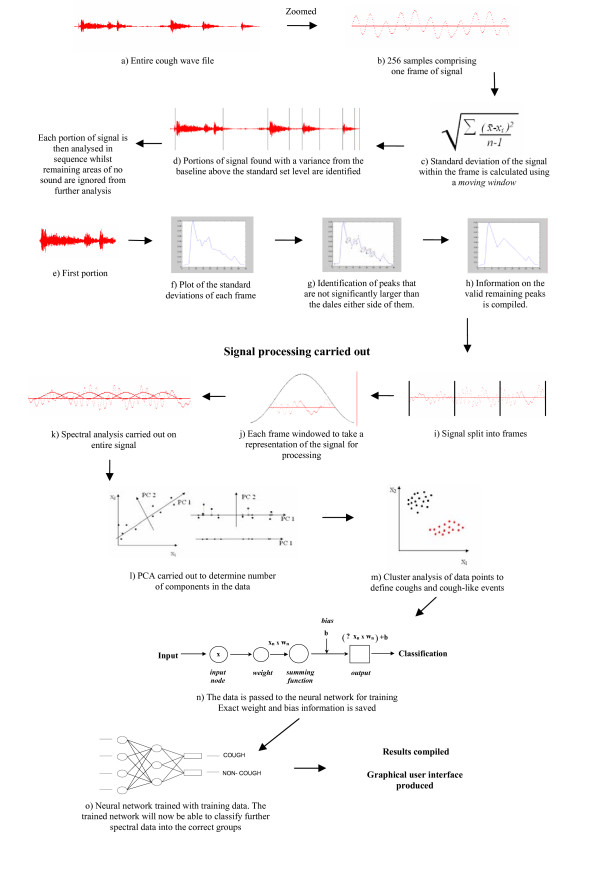
Pattern Recognition Approach to cough/non-cough classification.

Figure 1 shows a schematic representation of the HACC operation. Table 1 defines the variables and symbols used in the analysis.

**Table 1:** Symbols used and their settings.

Symbol	Meaning	Value
*f*_*s*_	Sampling Frequency	11025 Hz
*t*	Time in milliseconds	
σ_signal_	Windowed standard deviation of signal	Calculated as a function of time
Δ*t*_background_	Background interval	11026 points (1000 ms)
*thresh*_peak_	High (event detection) threshold	10 (×σ_background_)
*thresh*_limits_	Low (event start and end) threshold	2 (×σ_background_)
σ_background_	Standard deviation of background	
*N*_train_	Number of reference patterns	150 (75 cough/75 non-cough)
*n*_b-o-f_	Number of mel bank-of-filters cepstral coefficients	42 (14+14 1^st ^derivatives +14 2^nd ^derivatives)
*n*_LPC_	Number of LPC cepstral coefficients	14 (no derivatives)
*N*_cepstral_	Total number of cepstral coefficients (*n*_B-O-F _+ *n*_LPC_)	56
*N*_PCA_	Reduced number of features	45

Settings are based on established values and preliminary experiments. Symbols only used locally are explained in the text.

The first step is the isolation of sound events, as shown in Figure 1 (a to h).

**Figure 2:**
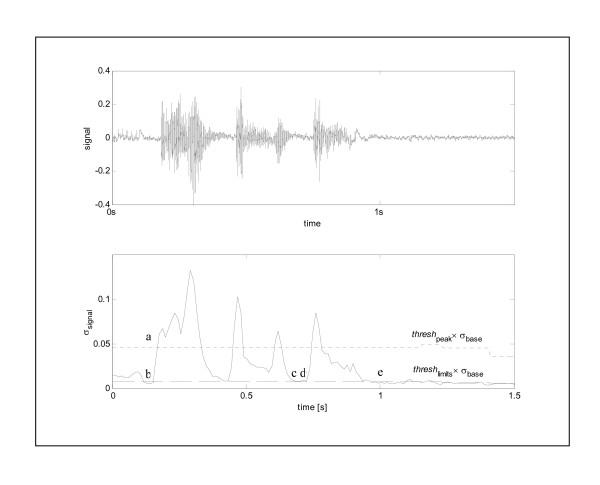
Sound detection. The top graph shows the original sound signal. In the bottom graph depicts σ_signal _and the two baseline threshold lines in which *thresh*_peak _= 10 and *thresh*_limits _= 1.5. Point 2(a) indicates the first standard deviation larger than *thresh*_peak _× σ_background_. Points 2(b) and 2(c) are the points nearest to point 2(a) where σ_signal _is smaller than *thresh*_limits _× σ_background_. The whole region between points 2(b) and 2(c) is a sound event. In the same way, the region between points 2(d) and 2(e) will be detected as a sound event.

The audio recording is initially converted into a 44.1 kHz 16 bit mono Microsoft digital wave file. For this process, the sound recordings are analysed at a sampling frequency of 11.025 kHz. The signal is then analysed using the moving windowed signal standard deviation σ_signal_, i.e. the standard deviation as a function of time. The moving window works along the entire length of the audio signal, taking each frame as the centre of a new window. This windowed standard deviation is similar to the more commonly used root mean square signal however, it corrects for deviations of the mean from zero. Portions of the signal containing no sound events will show a reasonably constant background signal (baseline) with small deviation relating to the inherent noise present in the signal. A sound event will cause the signal to rise above the baseline with a magnitude proportional to the validity of the signal. The moving window technique ensures the standard deviation of the background signal is not fixed for the duration of the signal; instead σ_background _at time *t *is calculated as the minimum σ_signal _between the start of the window, *t *- Δ*t*_background _and the end of the window, *t *+ Δ*t*_background_. Sound events are thus detected when σ_signal _for a particular window exceeds the threshold value, *thresh*_peak_, multiplied by σ_background _for that window. Although this procedure means that sound sensitivity varies to a certain extent, it allows for peak detection in noisy backgrounds. The start and end values of a sound event are defined as the nearest σ_signal _before and after the peak maximum which are below the defined low level calculated by *thresh*_limits _× σ_background_. Portions of the signal that are below this low level are removed and excluded from further analysis (Figure 2). The amount of noise within the section of signal is then reduced by smoothing. The standard deviations for each frame in the section are plotted and treated as a series of peaks. Peaks with variations lower than the noise-level are removed. The remaining frames of signal are compiled for signal processing.

The second step is the characterisation of sound events using a signal processing step as shown in Figure 1 (i to k). The sound events identified by analysis of the signal are then characterised. Each window undergoes a parameter measurement step in which a set of parameters is determined and combined into a test pattern (termed a feature vector). Because windowing is used, multiple test patterns are created for a single sound event. These test patterns are compared with a set of *N*_train _reference patterns for which the cough/non-cough classification is known. Depending on whether the test patterns are more similar to the cough or the non-cough reference patterns the corresponding sound event is classified as a cough or non-cough event respectively.

The third step is pattern comparison and decision-making as shown in Figure 1 (l to o). For this HACC uses a PNN. This network provides a general solution to pattern classification problems by following a Bayesian classifiers approach. The PNN stores the reference patterns.

Instead of classifying single patterns, HACC classifies complete sound events. The *p*_*k *_values for all test patterns belonging to the sound event are summed yielding a sum of probabilities ∑*p*_*k *_for each class *k*. The sound event is classified as a member of the class with the largest ∑*p*_*k*_.

### Manual cough recognition and counting

**Figure 3:**
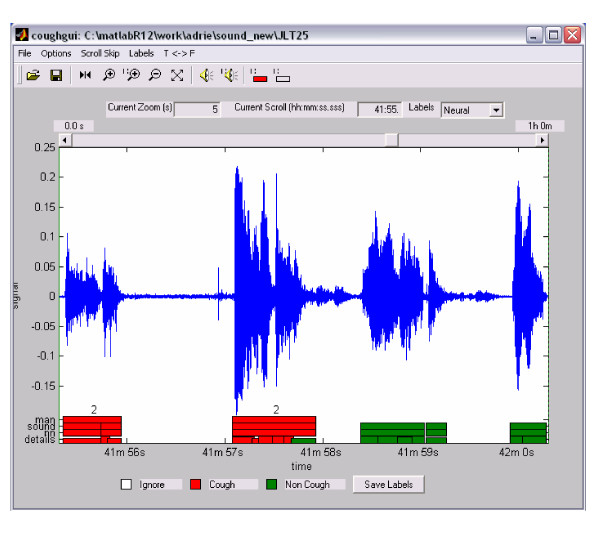
Graphical User Interface (GUI) for human listener.

In order to create and test the HACC program, reference measurements are required. For this purpose a graphical user interface (GUI) was developed (see Figure 3). This GUI lets the user scroll through a recording while displaying the corresponding waveform. The displayed sound can be played and coughs can be identified.

### Creation of the reference patterns

Sound recordings from 23 subjects are used to create a set of 75 cough patterns and 75 non-cough patterns. The first step is to identify suitable cough and non cough events in all 23 recordings. Suitability is determined by the clarity of the sound, and by its ability to add relevant variation to the dataset. Non cough events are sounds present in the audio recording which are not coughs. These events are combined into a cough pattern matrix **X**_cough _(10324 cough patterns) and a non-cough pattern matrix **X**_non-cough _(254367 non-cough patterns). The length of the feature vectors in these matrices is reduced by performing a principal component analysis (PCA) [27]. The combined **X**_cough_, **X**_non-cough _matrix is first auto-scaled (scaling of the feature values to zero mean and unit variance [28,29]) then as defined by PCA, only the scores that describe more than 0.5% of the variance are used. Experimental data is scaled using the means and variances of the reference data and projected onto the principal component space using a projection matrix. The reference patterns used for creation of the PNN are obtained by performing two *k*-means [30] clusterings (*k *= 0.5*N*_train_) of approximately 2000 cough and non-cough patterns. The initial 2000 patterns are selected from **X**_cough _and **X**_non-cough_. The reference patterns are then passed through the PNN for future classification of cough and non-cough patterns.

For validation, one hour recordings of a further 10 subjects, not previously used in the creation of cough patterns, were analysed by two independent listeners (methods A and B) and HACC (+ listener for actual cough counting; method C). Listener A was an experienced cough counter that worked in the cough clinic, whilst listener B was a undergraduate project student with no experience of cough counting. Cough is defined as an explosive sound separated by a fall of sound level to below threshold. Thus, a peel of coughs is counted as a number of separate coughs. We have recognised that a small number of cough events occur with a double sound element of under one second duration and we have programmed HACC to recognise these and identify them to the operator who decides whether they wish to classify them as single or multiple coughs.

Currently, HACC identifies coughs and labels them, though as yet does not automatically count them. Therefore a further listener was also used in method C to count the labelled coughs using the GUI. Subsequently the GUI was used to definitively identify cough and non cough events in the recordings to establish HACC's sensitivity and specificity.

## Results

**Figure 4:**
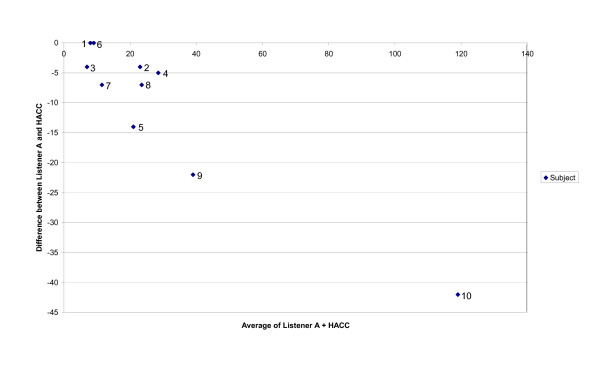
The Bland Altman plot showing the difference between the total number of coughs per subject as recorded by the experienced listener (A) compared to the HACC program (C).

Table 2 lists the total number of coughs reported by the human observers and HACC. The experienced observer frequently reported fewer coughs, mean 23.7 than either the inexperienced observer or HACC both 34.2, p ≤ 0.05. A Bland-Altman plot comparing the total number of coughs calculated by the experienced listener (A) and the HACC program (C) is shown in Figure 4.

**Table 2:** Counted coughs

	A	B	C
subject 1	8	6	8
subject 2	21	22	25
subject 3	5	6	9
subject 4	26	25	31
subject 5	14	30	28
subject 6	9	13	9
subject 7	8	8	15
subject 8	20	29	27
subject 9	28	53	50
subject 10	98	150	140

Mean	23.7	34.2	34.2

Using the GUI, the average sensitivity was calculated to be 0.80 with a range of 0.55 to 1.00 while the specificity was 0.96 with a range of 0.92 to 0.98. Using HACC it was possible to identify coughs in an hour long recording in an average time of 1 minute 35 seconds, a reduction of 97.5% in counting time.

Reproducibility of repeated HACC analysis is 100%.

The average percentage of false positives compared to true positives was calculated to be 20%. False positives were caused by similar sounds such as laughter, loud bangs and other subjects coughing.

## Discussion

The clinical importance of the analysis of continuous cough recording lies in the temporal pattern of cough events. Because of the episodic nature of cough, recordings must be undertaken for a prolonged period which until the development of automatic cough counting necessitated an equally long period of analysis. Our study introduces the technique of computerised cough sound analysis which dramatically reduces analysis time.

To optimally classify a sound event as cough or non-cough the feature vectors should obey certain requirements. The feature vectors of coughs from different subjects should have similar values. Non-cough events should give dissimilar feature vectors than cough events. The features should not be correlated and preferably follow a probability distribution that is well described as a sum of Gaussians. It is also desirable that the features do not depend on the sound amplitude; the cough loudness is not the same for different people and it makes the placement of the microphone less critical.

These requirements are very similar to those in speech recognition. To recognise speech, a reliable, robust and most widely used feature set based on frequency content are cepstral coefficients. Cepstral coefficients are the coefficients of the Fourier transform representation of the log magnitude spectrum. They are good at discriminating between different phonemes (speech sounds), are fairly independent of each other and have approximately Gaussian distribution for a particular phoneme. The cepstral coefficients are normally calculated via one of the following two pre-processing routes: linear predictive coding (LPC) or a bank-of-filters front-end processor [26, 31, 32].

The recognition performance can be improved by extending the representation with temporal cepstral derivative information. Cepstral derivatives are obtained by the published method [32]. The feature vectors used in HACC consist of a pre-treated combination of *n*_B-O-F _mel bank-of-filters cepstral coefficients with their first and second derivatives and *n*_LPC _LPC cepstral coefficients (without derivatives). Pre-treatment consists of scaling followed by projection into the principal component space obtained for the reference samples. This pre-treatment reduces the number of features in each pattern from N_cepstral_(= *n*_B-O-F _+ *n*_LPC _in this study 56) to *N*_PCA _(here 45).

The coughs in the cough events need to be counted by a human listener. For this purpose, the GUI is used. However, in this procedure only events classified as cough by HACC have to be listened to. This procedure yields a huge reduction in listening time (mean 97.5%) compared to human counting. Our aim is to improve HACC in the near future so that human counting is no longer necessary.

The results show a significant increase in the number of coughs reported by the inexperienced listener and HACC compared to those reported by the experienced listener. This difference is caused by both the inexperienced listener and HACC detecting and counting coughs from sources other than the subject under study. The subjects were recorded in a clinic alongside other patients and as a result, other coughs are clearly audible on the recordings. The inexperienced listener B and HACC simply counted all audible coughs which explains why the data from B and C are so similar, and exaggerated. Clearly the experience of listener A discerns between the subject closest to the microphone and the other cough events that are audible on the recordings. Thus it is clear that even with this slight disparity between the computer and the experienced listener, the computer has in fact classified all the coughs on the recordings, but without any distinction as to the source of the coughs.

Since HACC is not subject-specific in its cough classification, improving the counting accuracy is best achieved by excluding the non-subject coughs from the recording. Using a different microphone with a lower sensitivity will ensure only high-amplitude sounds occurring close to the microphone will be detected, thus discerning the subject's coughs from ambient coughs. This modification will also diminish problems with background noise. The recordings for this study were all made in a similar environment, with the subjects ambulatory and television and conversation freely permitted. The use of a lower sensitivity microphone will help to diminish background noise before any processing by HACC is carried out.

For the development of HACC processing, hour long recordings of each subject were made, it was felt that this duration of recordings contained a sufficient number of cough and non-cough events to carry out an assessment of the system.

Future work will test HACC's ability to process much longer duration of recordings containing a wider variety of patient groups.

One of the major advantages of the automated recording is that it is possible to re-analyse the data with minimal effort and achieve consistent results. Thus, when the same recordings were reprocessed the events classified as coughs in one run were also found to be coughs in subsequent runs. This allows development of a statistically stable analysis method with a known statistical confidence limit on the results.

## Conclusion

An automated system for the analysis of sound files containing coughs and other non-cough events has been developed, with a high robustness and good degree of accuracy towards the number of actual coughs in the audio recording. Although HACC is unable to distinguish between coughs of the subject under study and ambient coughs, changes to the hardware could resolve this problem in the future.

## Declaration of competing interests

The author(s) declare that they have no competing interests.

## Authors' contributions

AD developed the HACC program, processed the recordings and was the listener to obtain results for HACC. AD also drafted the manuscript.

AM designed and coordinated the clinical studies and assisted with the manuscript.

AW and SB developed the manuscript.

All authors read and approved the final manuscript.
